# Sensitivity and specificity analyses of COVID-19 screening protocol for emergency medical services: A STARD-compliant population-based retrospective study

**DOI:** 10.1097/MD.0000000000030902

**Published:** 2022-10-07

**Authors:** Hidetada Fukushima, Yuichi Nishioka, Kei Kasahara, Hideki Asai, Shota Sonobe, Tomoaki Imamura, Shigeo Muro, Kenji Nishio

**Affiliations:** a Department of Emergency and Critical Care Medicine, Nara Medical University, Kashihara City, Nara, Japan; b Department of Diabetes and Endocrinology, Nara Medical University, Kashihara City, Nara, Japan; c Department of Public Health, Health Management and Policy, Epidemiology, Nara Medical University, Kashihara City, Nara, Japan; d Center for Infectious Disease, Nara Medical University, Kashihara City, Nara, Japan; e Division of Intensive Care, Nara Medical University, Kashihara City, Nara, Japan; f Department of Respiratory Medicine, Nara Medical University, Kashihara City, Nara, Japan; g Department of General Medicine, Nara Medical University, Kashihara City, Nara, Japan.

**Keywords:** coronavirus, emergency medical services, epidemiologic factors, screening, sensitivity and specificity, symptom assessment, vital signs

## Abstract

During the novel coronavirus disease (COVID-19) pandemic, emergency medical services (EMS) has borne a huge burden in transporting emergency patients. However, the protocol’s effect on identifying emergency patients who are likely to have COVID-19 is unknown. We aimed to evaluate the diagnostic accuracy of a prehospital COVID-19 screening protocol for EMS.

We conducted this population-based retrospective study in Nara Prefecture, Japan. The Nara Prefectural Government implemented a screening protocol for COVID-19 comprising the following symptom criteria (fever, cough, sore throat, headache, malaise, dysgeusia, or anosmia) and epidemiological criteria (contact history with confirmed COVID-19 cases or people with upper respiratory symptoms, or travel to areas with high infection rate). A patient meeting at least one criterion of each class was considered positive. We evaluated all 51,351 patients from the regional EMS database of the Nara Prefecture (emergency Medical Alliance for Total Coordination of Healthcare) who were registered from June 15, 2020 to May 31, 2021 and had results of COVID-19 reverse transcription polymerase chain reaction (RT-PCR) tests. We assessed the sensitivity, specificity, positive predictive value (PPV), and negative predictive value (NPV) of this protocol. We also assessed how these outcomes changed by adding vital signs and conducted a 10-fold and 100-fold prevalence simulation.

The screening protocol was used for 246/51351 patients (0.5%). Among them, 31 tested positive after EMS transportation. This protocol’s sensitivity, specificity, PPV, and NPV were 40.8%, 99.6%, 12.6%, and 99.9%, respectively. With the addition of ≥2 vital signs (body temperature ≥37.5 °C, respiratory rate ≥20 breaths/minute, and oxygen saturation <90%), sensitivity and PPV changed to 61.8% and 1.0%, respectively, while NPV remained 99.9%. With a 10-fold and 100-fold increase in disease, the protocol PPV would be 59.0% and 94.3%, and NPV would be 99.1% and 90.7%, respectively, and with additional vital signs, PPV would be 8.9% and 53.1%, and NPV would be 99.4% and 93.2%, respectively.

This COVID-19 screening protocol helped enable EMS transport for patients with COVID-19 with a PPV of 12.6%. Adding other vital sign variables may improve its diagnostic value if the prevalence rate increases.

## 1. Introduction

Since the first report of severe idiopathic pneumonia on November 22^nd^, 2019, in Wuhan, China, the world’s population has been dealing with severe acute respiratory syndrome coronavirus 2 (SARS-CoV-2) infection, causing the coronavirus disease (COVID-19) pandemic. As of August 2022, the number of patients who had COVID-19 was more than 580 million, and the number of deaths had surpassed 6 million worldwide.^[1]^ The number of patients with COVID-19 in Japan continues to rise despite the government’s attempts to control the pandemic with several state of emergency declarations.

Because the virus is highly contagious, most healthcare facilities in Japan were not adequately prepared in the early stages of this pandemic. Thus, there was a shortage of diagnostic regimes, protective equipment, patient admission strategies, and isolation beds.^[2]^

Public and private emergency hospitals started to deny admission to patients who were feverish or experienced upper respiratory symptoms and were transported by emergency medical services (EMS) because of this unpreparedness to receive patients with COVID-19.^[2,3]^ This situation arose in many regions of Japan. In Nara Prefecture, a local prefecture with a population of 1.3 million, EMS agencies also faced difficulties finding hospitals to transport patients, regardless of whether they were suspected of having COVID-19 or not. Therefore, Nara officials designated certain local hospitals to receive patients who were highly suspected of having COVID-19. However, the definitive identification of COVID-19-positive cases during the prehospital phase is impossible as the general symptoms of this infectious disease are atypical: fever, cough, and/or general malaise. Patients known to have visited an area where new positive cases have been increasing met with individuals who tested positive or had a family member who tested positive are highly likely to be positive themselves: this can be the only available criteria in distinguishing between patients suspected to have COVID-19 and those who have a fever for a different reason. Officials developed a screening protocol comprising clinical symptoms and the abovementioned contact epidemiological information based on advice from medical experts, including infectious disease specialists. They implemented this protocol for EMS to enhance the division of emergency patients into suspected COVID-19 cases and others. Since few studies have addressed the effect of a screening protocol that can help EMS distinguish patients with from those without COVID-19, we aimed to estimate the diagnostic accuracy of this screening tool for suspected COVID-19 cases for EMS during the pandemic in Nara Prefecture.

## 2. Methods

### 2.1. Study design

We conducted this retrospective, population-based, descriptive study of regional emergency records. This study was approved by the institutional ethics board of Nara Medical University (IRB No. #2793). The need for informed consent was waived by the institutional board because of the retrospective nature of the study.

### 2.2. Study region

Nara Prefecture is one of 47 prefectures in Japan, with a population of 1.3 million and an area of 3690 km^2^, bordering Osaka and Kyoto prefectures. It contains 3 tertiary referral hospitals.

The EMS in Nara Prefecture consists of 3 fire departments: Nara City Fire Bureau, Ikoma City Fire Department, and Nara Wide Area Fire Department. The EMS of these departments responds to approximately 67,000 calls per year.

The first human-to-human COVID-19 transmission case in Japan was identified on January 28, 2020, in Nara Prefecture. Thereafter, no new COVID-19 cases were reported until March 6. From the second case, the number of COVID-19 cases slowly accumulated but remained below 100 until the Japanese Government lifted the state of emergency. Since July 4, 2020, the number of positive cases has increased more rapidly. As of May 31, 2021, the cumulative number of COVID-19 cases in Nara Prefecture was 7891.

### 2.3. COVID-19 screening protocol for emergency patients

The Nara Prefectural Government certified public medical institutions as hospitals that had to receive patients with or suspected to have COVID-19. To identify emergency cases with a high probability of COVID-19 positivity, the Prefectural Government implemented a COVID-19 screening protocol. This protocol was developed based on advice from medical experts, including infectious disease specialists, incorporating clinical symptoms and epidemiological information. Clinical symptoms included being feverish (body temperature not specified), having sore throat, rhinorrhea, cough, sputum, general malaise, anosmia, or dysgeusia. These symptom criteria were included from the preliminary epidemiological study on 770 cases between January 25 and May 6, 2020, in Japan, showing that the majority of cases presented the above symptoms. Epidemiological criteria were as follows: contact with an individual who tested positive for SARS-CoV-2, having visited within 2 weeks an area in which numbers of new COVID-19 cases had been rapidly increasing (e.g., Tokyo or Osaka), and having had contact within 2 weeks with individuals who presented with any of the above clinical symptoms. These epidemiological criteria were developed based on the airborne transmission manner of SARS-CoV-2 and its incubation period of up to 14 days. A patient with one or more clinical symptoms who met at least one epidemiological criterion was highly suspected of having COVID-19 and was transported to the designated hospitals.

The fire departments adopted this screening protocol for emergency patients effective on June 15, 2020. Transported patients under infectious disease or public health specialists’ supervision underwent reverse transcription polymerase chain reaction (RT-PCR) tests when a physician considered them likely to be patients with COVID-19. If a patient tested positive for SARS-CoV-2 by an RT-PCR test, the local health center announced the result not only to the patient and the hospital but also to the EMS that transported the patient. These notifications were to be sent to those with possible contact within a period of potential transmission.^[4]^

### 2.4. Study population

We included all emergency patients who were transported by EMS during the study period from June 15, 2020, to May 31, 2021. Patients known to be COVID-19 positive and those who underwent interfacility transfer were excluded.

### 2.5. Data extraction

EMS records were extracted mainly from an EMS database called “emergency Medical Alliance for Total Coordination of Healthcare (e-MATCH).” It is an online system that is navigable on portable tablets and assists EMS in evaluating patients and finding the closest appropriate hospital. Prehospital records, including the patient’s vital signs, such as body temperature, pulse rate, respiratory rate, and degree of oxygen saturation as determined by pulse oximetry (SpO_2_), are stored in the e-MATCH database and are available only to authorized personnel. Data of patients suspected to have COVID-19 following the screening protocol and those with positive RT-PCR results for COVID-19 were obtained from the prehospital records of each fire department.

### 2.6. Data linkage

Each fire department issues ID numbers for e-MATCH datasets and prehospital records. Data linkage was performed by matching those IDs. Where IDs were missing, data linkage was performed using the time stamp of the emergency dispatch, which is issued automatically within the system.

### 2.7. Exclusion criteria

We excluded patients with missing or erroneous data of vital signs variables. We defined erroneous data as follows: body temperature below 33.5 or over 42.2 °C, respiratory rate less than 10 or more than 51 breaths/minute, heart rate less than 40 or over 225 beats per minute, and SpO_2_ less than 70%.

### 2.8. Primary and secondary outcomes

The primary outcome was this protocol’s sensitivity, specificity, and positive predictive value (PPV). Patients were deemed to have or not have COVID-19 based on RT-PCR test results. Secondary outcomes were analysis of EMS time intervals and the number of calls for hospital admission in cases that met screening protocol (suspected group) to those that did not (not-suspected group).

### 2.9. Statistical analysis

Descriptive statistics were used to characterize the study population. We also simulated the effectiveness of the protocol as the prevalence increased. The desired sample size was calculated for an absolute precision of 1% (that is, 10,000 individuals). The candidate population included patients suspected of having COVID-19, as identified based on an algorithm (COVID-19 screening protocol with other variables [heart rate, respiratory rate, body temperature, and oxygen saturation]). We computed the sensitivity, specificity, PPV, negative predictive value (NPV), prevalence, kappa value, and Youden index for each simulated algorithm according to sex, age group, and Japanese academic years completed (from April to March), with corresponding 95% confidence intervals (CIs). The sensitivity and specificity were the probabilities of each algorithm correctly identifying patients with and those without COVID-19, respectively. The PPV was the proportion of those identified with each algorithm as having COVID-19 who were COVID-19 positive. The NPV was the proportion of those identified with each algorithm as not having COVID-19 who were COVID-19 negative. Prevalence estimates were calculated for each algorithm. A kappa statistic was calculated for the agreement between each algorithm and the reference standard to identify the algorithms that maximize the kappa statistic.^[5]^ The Youden index was calculated to give equal weight to sensitivity and specificity, as follows: (sensitivity + specificity)–1. Categorical variables are described as numbers with percentages, and continuous variables are expressed as medians with interquartile ranges. Statistical significance was set at *P* < .05. All statistical analyses were performed with IBM SPSS Statistics for Windows ver. 25.0 (IBM Corp., Armonk, NY, USA).

## 3. Results

**Figure 1.**
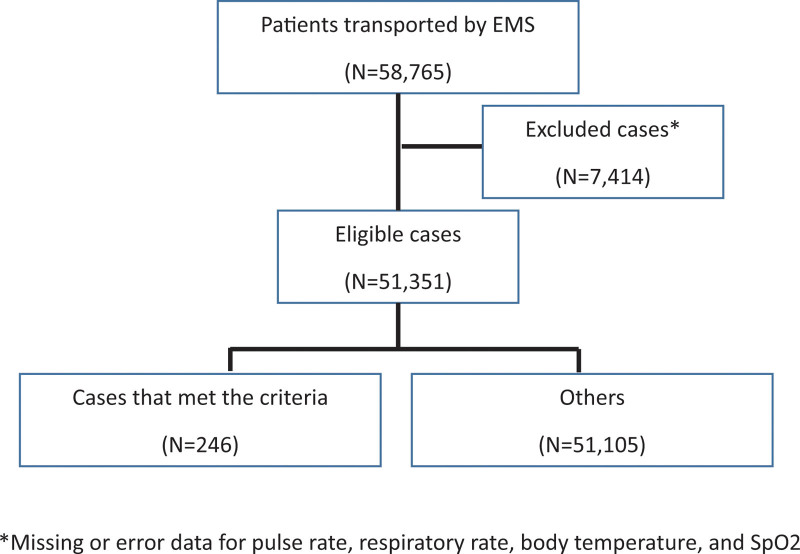
Study population.

Among 58,765 patients transported by EMS during the study period, 51,351 eligible patients underwent further analysis. Among them, 246 patients fulfilled the screening criteria, and 51,105 were considered to have a low probability of having COVID-19 (Fig. 1).

*Excluded because of missing or erroneous vital sign data. EMS = emergency medical services.

**Figure 2.**
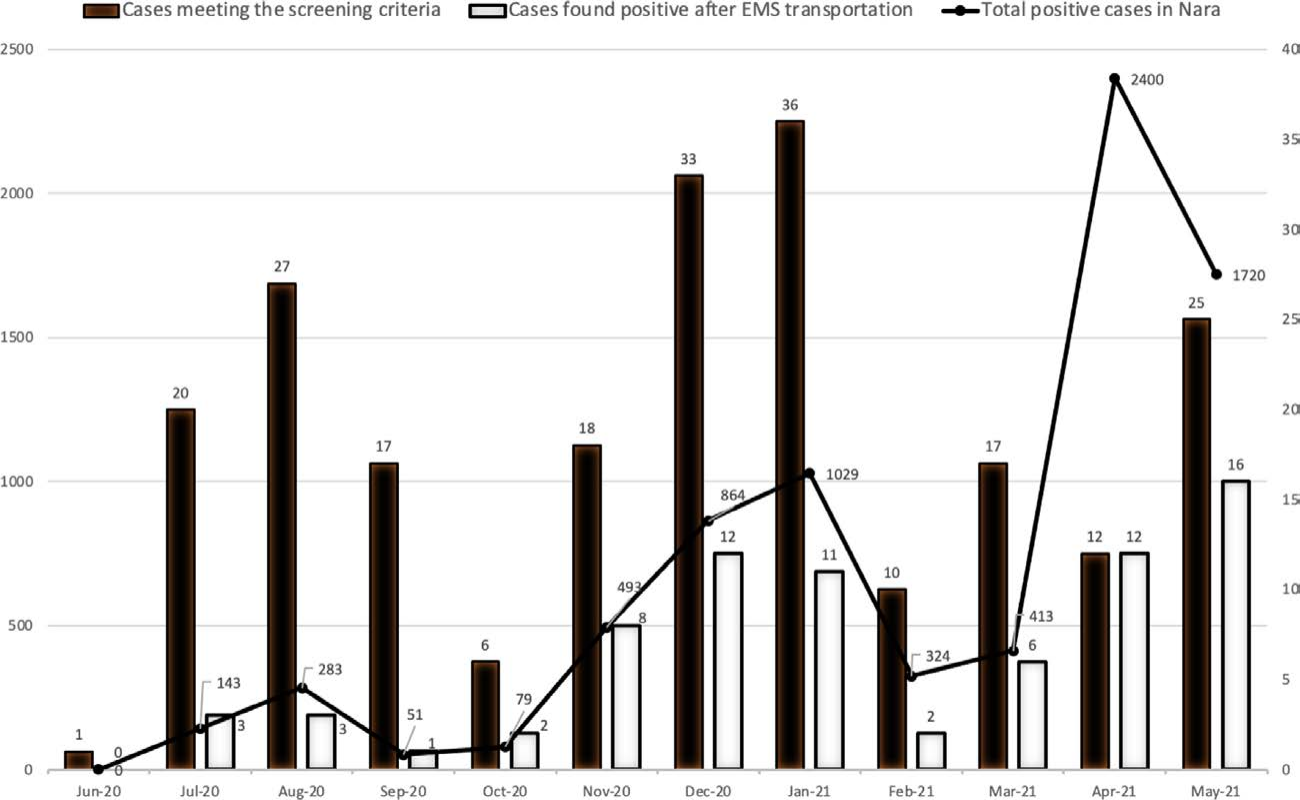
COVID-19 during the study period in Nara Prefecture, Japan. Patients that met the screening criteria (solid bars) and patients diagnosed with COVID-19 after emergency medical services (EMS) transportation (blank bars). The line illustrates the trend of COVID-19 cases in Nara Prefecture during the study period. COVID-19, coronavirus disease.

Figure 2 illustrates the trends of COVID-19-positive cases in Nara Prefecture during the study period. The number of patients who tested positive after EMS transportation increased along with the trend of positive cases in the entire prefecture, while the number of patients who met the screening protocol did not follow this trend. The number of patients who met the screening criteria did not increase in April. This may be due to the temporary impact of news reports on emergency transportation to hospitals requiring long hours.

COVID-19 = coronavirus disease.

Table 1 summarizes the characteristics of the study participants. The suspected group was younger than the not-suspected group (median age: 55 years vs 73 years, *P* < .001). Each EMS time interval tended to be longer in the suspected group, and the proportion of cases requiring 2 or more calls to ensure admission to the hospital was higher in this group, indicating that accredited hospitals were accepting many other patients with COVID-19 from home and non-designated hospitals. Conversely, the not-suspected group was transported to hospitals smoothly.

**Table 1:** Characteristics of the study population.

		Suspected group		Not-suspected group		*P* values
		n = 246	Missing cases, (n)	n = 51,105	Missing cases, (n)	
Age, y		55 (26–80)	0	73 (51–84)	3134	<.001
Male, n (%)		126 (51.2)	0	23845 (46.7)	3125	.647
EMS time intervals						
	Response time, min	10 (8–14)	1	9 (7–11)	85	<.001
	On-scene time, min	21 (14–34)	1	17 (12–23)	122	<.001
	Transportation time, min	11 (7–17.3)	1	10 (6–15)	53	.032
	Duration of calls to hospitals, min	9 (5–20.0)	0	6 (4–10)	408	<.001
	Calls by EMS until hospital acceptance	1 (1–2)	0	1 (1–1)	44	<.001
	Cases by EMS required more than 2 calls for hospital acceptance, n(%)	76 (30.9)	0	10244 (20.1)	124	<.001
Systolic blood pressure, mm Hg		130 (116–148.5)	5	141 (120–163)	1784	<.001
Diastolic blood pressure, mm Hg		78 (68.8–90)	8	80 (70–94)	3561	.022
Heart rate, bpm		100 (87–114)	0	88 (75–100)	0	<.001
Respiratory rate, breaths/min		20 (20–24)	0	20 (18–24)	0	<.001
Body temperature		38.0 (37.0–39.0)	0	37.0 (36.0–37.0)	0	<.001
Cases with body temperature of ≥37.5 °C, n (%)		156 (63.4)	0	7163 (14.0)	0	<.001
SpO_2_, %		97 (95–98)	0	97 (96–99)	0	<.001
Cases with positive result for COVID-19 RT-PCR, n (%)		31 (12.6)	0	45 (0.1)	0	<.001

Values are indicated as median (range), unless otherwise indicated. SpO_2_, pulse oximetry.

COVID-19 = coronavirus disease, EMS = emergency medical services, RT-PCR = reverse transcription polymerase chain reaction.

Regarding vital signs obtained by EMS on the scene, heart rate and body temperature were higher in the suspected group (median heart rate: 100 bpm vs 88 bpm, *P* < .001; median body temperature: 38 °C vs 37 °C, *P* < .001). Among the 246 patients in the suspected group, 31 (12.6%) had positive RT-PCR results for COVID-19, while 45 (0.08%) patients had positive results in the not-suspected group.

Table 2 summarizes the accuracy of this study’s screening protocol. The screening protocol’s sensitivity, specificity, PPV, and NPV were 40.8%, 99.6%, 12.6%, and 99.9%, respectively. We assessed the change in sensitivity and specificity following the addition of other variables such as body temperature ≥37.5 °C, respiratory rate ≥20 breaths/minute, and SpO_2_ < 90%. We discovered that patients fulfilling the original screening criteria and those who met 2 or more of these additional variables yielded a sensitivity of 61.8% and a specificity of 90.7%. However, the PPV decreased from 12.6% to 1.0% with the addition of these variables, while the NPV remained 99.9%.

**Table 2:** Accuracy of algorithms to identify patients with COVID-19.

Algorithm	TP	TN	FN	FP	Sensitivity (%)	95% CI		Specificity (%)	95% CI		PPV (%)	95% CI		NPV (%)	95% CI		Prevalence estimate	Kappa	Youden
Screening protocol	31	51,060	45	215	40.8%	29.7%	51.8%	99.6%	99.5%	99.6%	12.6%	8.5%	16.7%	99.9%	99.9%	99.9%	0.00	0.19	0.40
Screening protocol with vital signs criteria	47	46,483	29	4792	61.8%	50.9%	72.8%	90.7%	90.4%	90.9%	1.0%	0.7%	1.2%	99.9%	99.9%	100.0%	0.09	0.02	0.52
10-Fold prevalence simulation																			
Algorithm	TP	TN	FN	FP	Sensitivity (%)	95% CI		Specificity (%)	95% CI		PPV (%)	95% CI		NPV (%)	95% CI		Prevalence estimate	Kappa	Youden
Screening protocol	307	50,385	446	213	40.8%	37.3%	44.3%	99.6%	99.5%	99.6%	59.0%	54.8%	63.3%	99.1%	99.0%	99.2%	0.01	0.48	0.40
Screening protocol with vital signs criteria	466	45,840	287	4757	61.8%	58.4%	65.3%	90.6%	90.3%	90.9%	8.9%	8.1%	9.7%	99.4%	99.3%	99.4%	0.10	0.13	0.52
100-Fold prevalence simulation																			
Algorithm	TP	TN	FN	FP	Sensitivity (%)	95% CI		Specificity (%)	95% CI		PPV (%)	95% CI		NPV (%)	95% CI		Prevalence estimate	Kappa	Youden
Screening protocol	3072	43,635	4459	185	40.8%	39.7%	41.9%	99.6%	99.5%	99.6%	94.3%	93.5%	95.1%	90.7%	90.5%	91.0%	0.06	0.53	0.40
Screening protocol with vital signs criteria	4658	39,699	2874	4120	61.8%	60.7%	62.9%	90.6%	90.3%	90.9%	53.1%	52.0%	54.1%	93.2%	93.0%	93.5%	0.17	0.49	0.52

Reference standard: the Specific Health Checkups in Japan (N = 165,515); total patients = 2,999,152.

95% CI = 95% confidence interval, CI = confidence interval, FN = false negative, FP = false positive, Kappa = kappa index, NPV = negative predictive value, PPV = positive predictive value, Prevalence estimate = prevalence of patients with screening positive, TN = true negative, TP = true positive, Youden = youden index,

*Respiratory rate of 20 or more breaths/min., Body temperature of 37.5 or more degree Celcius, less than 90% of SpO_2_ values.

We simulated the change in PPV and NPV as the disease prevalence increased. With a ten-fold increase in prevalence, the PPV of the study protocol improved to 59.0%, while NPV remained at 99.1%. The PPV improved from 1.0% to 8.9% with two or more additional variables, while the NPV remained almost the same (99.4%). If the prevalence was 100 times higher, the PPV increased to 94.3%, and the NPV decreased to 90.7%. With 2 or more additional variables, the PPV improved to 53.1%, while the NPV increased slightly to 93.2%.

## 4. Discussion

The COVID-19 screening protocol helped EMS to identify patients with a high probability of having COVID-19, with a PPV of 12.6%, in the emergency setting in Japan, where the prevalence of new coronavirus infections is 0.1 to 0.2%. Although the sensitivity of this screening tool alone was only 40.8%, this could be improved to 61.8% by adding physical findings: respiratory rate ≥20 breaths/minute, body temperature ≥37.5 °C, and SpO_2_ < 90%, while specificity and PPV decreased.

The COVID-19 pandemic has created many obstacles to EMS systems in many regions of the world. In several observational studies, many patients were reportedly not transported to hospitals or the temporary emergency departments of hospitals were closed.^[6–8]^ When medical facilities were insufficient and unprepared at the beginning of the pandemic, as in Japan, it was critical for EMS to screen emergency patients and transport patients that were highly suspected of having COVID-19 to accredited hospitals and other patients to non-accredited emergency hospitals. This was especially important in Japan, where the emergency medical system is stratified into 3 levels based on the severity of the patient’s condition. Generally, primary and secondary hospitals in Japan, which treat patients with minor to moderate emergencies, are not designated for emerging infectious diseases such as COVID-19. At the early stage of this pandemic, there were only 2 medical facilities that could admit patients who were suspected of having COVID-19 in Nara Prefecture. Thus, conditions were ripe for disruption, confusion, or uncertainty in EMS and patient care alike until other local emergency hospitals were equipped with COVID-19 testing devices and personal protective equipment. Our study results suggest that the screening protocol assisted EMS in sorting patients based on suspected COVID-19 during this pandemic. At the same time, other emergency patients who were unlikely to be positive were transported to hospitals without difficulties compared to those who were positive when screened.

There are only a few population-based reports on EMS decisions on the transport of patients suspected to have COVID-19. Fernandez et al^[9]^ reported that the EMS decision had a sensitivity of 78% and a PPV of 20%. However, the reported decisions were mainly based on the impression of the EMS in charge. Regarding specific symptoms or clinical signs that are strongly related to confirmed COVID-19-positive cases, Saegerman et al^[10]^ investigated 11 symptoms in patients who presented to the emergency department (dyspnea, chest pain, rhinorrhea, sore throat, dry cough, wet cough, diarrhea, headache, myalgia, fever, and anosmia). Among these, they reported that fever (odds ratio: 3.66, 95% CI: 2.97–4.50) and dry cough (odds ratio: 1.71, 95% CI: 1.39–2.12) were strongly associated with a COVID-19 diagnosis, which is consistent with the results of another previous report.^[11]^ In contrast, others have reported that EMS encounters about 30% of those who were later confirmed to have COVID-19 but have no symptoms of fever, cough, or shortness of breath.^[12]^ These upper respiratory symptoms are nonspecific and may not be sufficient for EMS to identify patients with COVID-19. Other critical information in the identification of infectious diseases include the patient’s contact history with individuals confirmed to be infected. Epidemiological information, such as whether a patient visited areas where the number of COVID-19 cases is increasing and whether a patient was in contact with individuals who were confirmed/suspected to have COVID-19, can be helpful. Lara et al^[11]^ reported that the odds ratio of such epidemiological information for COVID-19 was 2.47 (95% CI: 1.29–4.70), comparable to that of fever (odds ratio: 3.63, 95% CI: 1.93–6.85). However, this information may not be available, especially when the prevalence rates increase. Thus, a high prevalence rate may increase the number of false-negative cases.

Our study results showed that the addition of clinical signs such as body temperature ≥37.5 °C, respiratory rate ≥20 breaths/minute, and SpO_2_ < 90% can improve the sensitivity of the screening protocol. Although these signs are also nonspecific,^[11,13,14]^ positive COVID-19 cases are likely to present with tachycardia, tachypnea, and/or low SpO_2_.^[9]^ By adding 2 or more of these additional variables, the sensitivity of the screening protocol increased, while the specificity and PPV decreased. As PPV is strongly affected by the prevalence rate, we also simulated 10-fold and 100-fold increases in the prevalence rate and discovered that the PPV increased as the prevalence rate increased. However, diagnostic criteria always involve a trade-off between sensitivity and PPV; when sensitivity increases, the PPV decreases, and the number of false-positive results increases, which may increase the workload on healthcare systems. Our results show that when the prevalence is low, as it was in Japan during the study period, this screening protocol is useful for EMS to classify both patients likely and unlikely to be COVID-19 and to transport these cases to designated hospitals without any difficulties. However, as the prevalence rate increases, vital signs should be incorporated into the protocol.

## 5. Limitations

One of this study’s limitations was its retrospective nature and the use of EMS data from only 1 region in Japan. We excluded 7414 cases because of missing vital sign data. These excluded cases accounted for more than 12% of the study population and, thus, may have affected the study results. Second, the spread of infection in the Nara Prefecture was relatively slow compared to that in big urban city areas. Therefore, caution should be exercised when interpreting our study results. EMS in urban areas may not be able to adopt this screening tool because of the high number of COVID-19 and other emergency cases. Third, not all the study participants underwent RT-PCR tests for SARS-CoV-2. During the study period, RT-PCR tests were not fully available and were performed only on possible patients with COVID-19. Most of the tests were performed at the discretion of physicians supervised by infectious disease or public health specialists. All the RT-PCR tests were performed in accordance with notifiable diseases surveillance for COVID-19 in Japan, and the number of positive cases has been officially recorded. Thus, we believe that RT-PCR tests during the study period were performed substantially and represents COVID-19 status with considerable accuracy.

Finally, hospital ambulances are not part of EMS in Japan. Irrespective of this, our study results may also be applied to any form of prehospital care setting.

## 6. Conclusions

Our study demonstrates the benefits of a COVID-19 screening protocol comprising both symptomatic and epidemiological criteria for use by EMS. To compensate for the lack of sensitivity, vital sign variables (body temperature ≥37.5 °C, respiratory rate ≥20 breaths/minute, SpO_2_ < 90%) can be added to the screening criteria, especially if the prevalence rate increases.

## Acknowledgments

We thank Nara City Fire Bureau, Ikoma City Fire Department, and Nara Wide Area Fire Department for providing data on study participants.

## Author contributions

H.F., H.A., and K.K. designed this study and collected data. H.F., Y.N., and T.I. performed statistical analysis. S.S., S.M., and K.N. supervised the findings of this work. All authors discussed the results and contributed to the final manuscript.

**Conceptualization:** Hidetada Fukushima.

**Data curation:** Hidetada Fukushima, Kei Kasahara, Hideki Asai.

**Supervision:** Shota Sonobe, Shigeo Muro, Kenji Nishio.

**Validation:** Yuichi Nishioka, Tomoaki Imamura.

**Writing – original draft:** Hidetada Fukushima, Kei Kasahara, Hideki Asai.

**Writing – review & editing:** Hidetada Fukushima, Kei Kasahara.
